# What Advantages the American Colleges of Dentistry Afford the Students and the Afflicted Public

**Published:** 1880-06

**Authors:** F. W. Rudolph

**Affiliations:** Hamburg, Germany.


### ARTICLE II.

### *What Advantages the American Colleges of Dentistry afford the Students and afflicted Public.*

A DISPUTATION BY DR. F. W. RUDOLPH, HAMBURG, GERMANY.

#### I.—DENTAL SURGERY IN GERMANY.

The time has not long passed, when Dental Surgery and the Dental Profession generally was looked upon as the step-child of the sciences. By and by in Germany, men of influence succeeded in securing for it its recognition as a branch of medicine, and on the part of the German government, a chair of Dental Surgery was established in some of the German Universities, and only one dentist appointed to lecture on this branch.

Of all the German Universities, only five have a chair for Dental Surgery, with one Docent each. So little is this important branch recognized that a small number of Dental Students attend.

In the whole German Empire, only six Dental Students graduated in the year 1879. The system of educating the German dentists has been of a very different kind. Before 1872, it may not be improper to state, that most everybody, who had a little preliminary education and attended to a few lectures, was admitted to a very simple dental examination of the State board, and when passed, was given the right to practice as a dentist. After this time, legislation in Germany elevated dentistry to a branch of medicine, and all those men with their very doubtful education who were in practice then, wanted to be recognized as members of the medical profession. Now every young man, who wants to study dentistry can only matriculate on the Universities when he has passed the first class (prima) of a High School, and then, he has to study for two courses before he is admitted to examination. At the university, he is compelled to hear every day for one hour, a lecture from the Dental Docent, and finally if one point is missing from the expected standard he will not be passed. This method of education is called by men of authority a very defective one.

The dentists took generally intelligent young men as apprentices in their laboratories, who stayed with them for three years, and then became Mechanical Dentists because they were not admitted to the State examinations. But, however, those who were able, went to America and studied Dental Surgery at the American Dental Colleges, with their Museums, Infirmaries, Laboratories, &c., and their excellent professors, and then came back to Germany as a perfect and competent dentist. But sorry to say, those graduated dentists in Germany are not disposed to recognize the American Doctors of Dental Surgery (this title though is recognized by the German Government when legal,) as their brethren and fellow-men, not as co-operators for the benefit of the suffering humanity, or representatives of the Dental profession, although the American Dentists do not pay much attention to this. With what disregard the German Dentist looks down on the American proves the memorial of Mr.

Mehlhardt, dentist, of Halberstadt, Germany, of which I have more to say afterwards.

In this memorial there is nothing said about the imperfect dental education in Germany, although a German Dental College is pleaded for, in it. The latter is just the thing that Germany needs, a chance to study, like America offers. Good dental professors and students will come in numerously. The six graduated dentists can not save the teeth of the suffering humanity, and as long as no good Dental College exists in Germany, whoever is able to do so, goes to America.

### II.—DENTAL SURGERY IN ENGLAND.

Dental Science has a very important place in England. There are in London two excellent institutions, after the American model for the education of dental students. In the future, everybody who wants to practice dentistry must procure previously the degree of Licentiate of Dental Surgery at the College, but also the American dentists are highly respected in England, and their offices are very much frequented by the public.

England pays close attention to all transactions and improvements in dentistry in America. This is another reason why America is looked upon as the mother of dentistry, and we don't need to agree with Mr. Mehlhardt when he says: "If young America has made the mistake, to allow everybody the practice of the healing arts, so this is to excuse as a mistake of youth." How generous!

### III.—DENTAL SCIENCE IN AMERICA.

The celebration of the golden wedding of his majesty the Emperor of Germany, gave occasion to Mr. Mehlhardt, dentist, of Halberstadt, Germany, to publish the already mentioned memorial and to dedicate it to the Emperor. In those pages, Mr. Mehlhardt uses very contemptible language about the American Dentists, and the American Colleges, thinking that only he is infallible, and considers a great number of his Colleagues as quacks and charlatans.

Besides other things, Mr. Mehlhardt asserts, that not the whole science of dentistry but only mechanical dentistry is taught at the American Colleges.

I have not only seen American dentists engaged at the operating chair, but have also heard scientific lectures from them, and must assert, that they have a better dental education than the greater number of German dentists can boast of. Concerning mechanical dentistry the Americans can not be beaten, and nobody will blame the public, when they seek the American dentists.

I have in my possession several of the catalogues and announcements of some of the best American Dental Colleges. For instance, Baltimore, her distinguished College, founded in 1839, a blessing for the dental student and suffering humanity. The dean of this College is one of the best Professors of Dental Pathology and Therapeutics in America, Professor Ferd. J. S. Gorgas, together with a faculty of five more distinguished Professors for Anatomy, Physiology, Chemistry, &c., &c., and excellent Demonstrators. The Baltimore Dental College has graduated already 881 Doctors of Dental Surgery, who are practicing all over the world, for the welfare of their patients.

But only Mr. Mehlhardt might cry about this, and despair. Why? Because this old gentleman believes that no American dentist is so competent and skilful as himself.

Furthermore, New York has an excellent dental College, of which Dr. Abbot is dean; also Boston, Philadelphia, &c. all of which are blessings to the world, and we only wonder, why Mr. Mehlhardt speaks with such absolute disregard about these institutions, and is not disposed to acknowledge anything that America has done for dentistry. Intelligent German dentists send their sons to America to study dentistry, and there to finish their education, and we only wish to have a like institution here in Germany. Because the first Medical authorities in Germany have stated that the present system of teaching dental students in Germany is a very defective one, and intelligent men always prefer the American Colleges.

These Colleges give the student all advantages to become perfect in his profession. They are equipped with museums, infirmaries, laboratories, &c., and their professors are men of the greatest ability in their chairs. The infirmaries are crowded with patients every day, and there the student can enjoy practice in full, and poor people are treated free of charge. We hope the time is not far distant when we will have in Germany an institution like them.

Mr. Mehlhardt says that he has enjoyed a large practice for thirty years. What was the state of dental surgery thirty years ago? What faculties; what chance for dental education; what dental literature, were then present? Certainly very insufficient ones. What immense progress is made during this time, and America is always ahead with its inventions and improvements.

What has Mr. Mehlhardt done in this 30 years for the progress of dentistry? He says the Medical Science, respecting dental surgery in America, is nothing but a system of swindling the public of their money. Further he says every toothache can be cured without the extraction of the tooth; all filling ought to be done free from any pain whatever to the patient. But how to do it, Mr. Mehlhardt keeps secret in his infallibility, but an American dentist would not hesitate to make such important discoveries known for the benefit of the suffering humanity. Thank them for it! That is true benevolence! That only is true help. Let us bring the American Colleges and their representatives a *Creseant! Vivant! Floreant!* May Germany have soon such an institution there.

---

[This article is dedicated most respectfully to F. J. S. Gorgas, A. M., M. D., D. D. S., Director of the College for Dentistry in Baltimore, by the author.]

